# Pto Kinase Binds Two Domains of AvrPtoB and Its Proximity to the Effector E3 Ligase Determines if It Evades Degradation and Activates Plant Immunity

**DOI:** 10.1371/journal.ppat.1004227

**Published:** 2014-07-24

**Authors:** Johannes Mathieu, Simon Schwizer, Gregory B. Martin

**Affiliations:** 1 Boyce Thompson Institute for Plant Research, Ithaca, New York, United States of America; 2 Department of Plant Pathology and Plant-Microbe Biology, Cornell University, Ithaca, New York, United States of America; Michigan State University, United States of America

## Abstract

The tomato—*Pseudomonas syringae* pv. *tomato (Pst)*—pathosystem is one of the best understood models for plant-pathogen interactions. Certain wild relatives of tomato express two closely related members of the same kinase family, Pto and Fen, which recognize the *Pst* virulence protein AvrPtoB and activate effector-triggered immunity (ETI). AvrPtoB, however, contains an E3 ubiquitin ligase domain in its carboxyl terminus which causes degradation of Fen and undermines its ability to activate ETI. In contrast, Pto evades AvrPtoB-mediated degradation and triggers ETI in response to the effector. It has been reported recently that Pto has higher kinase activity than Fen and that this difference allows Pto to inactivate the E3 ligase through phosphorylation of threonine-450 (T450) in AvrPtoB. Here we show that, in contrast to Fen which can only interact with a single domain proximal to the E3 ligase of AvrPtoB, Pto binds two distinct domains of the effector, the same site as Fen and another N-terminal domain. In the absence of E3 ligase activity Pto binds to either domain of AvrPtoB to activate ETI. However, the presence of an active E3 ligase domain causes ubiquitination of Pto that interacts with the domain proximal to the E3 ligase, identical to ubiquitination of Fen. Only when Pto binds its unique distal domain can it resist AvrPtoB-mediated degradation and activate ETI. We show that phosphorylation of T450 is not required for Pto-mediated resistance *in vivo* and that a kinase-inactive version of Pto is still capable of activating ETI in response to AvrPtoB. Our results demonstrate that the ability of Pto to interact with a second site distal to the E3 ligase domain in AvrPtoB, and not a higher kinase activity or T450 phosphorylation, allows Pto to evade ubiquitination and to confer immunity to *Pst*.

## Author Summary

Plant pathogenic bacteria inject effector proteins into plant cells to suppress immune responses and cause disease. The causal agent of bacterial speck, *Pseudomonas syringae* pv. *tomato,* is an important pathogen of tomato and a model system to study molecular plant-pathogen interactions. Here we report new insights into how the AvrPtoB effector can be recognized by the tomato kinase Pto to activate immunity. AvrPtoB is an active E3 ligase that is able to ubiquitinate host proteins and target them for degradation. The ability of Pto to resist ubiquitination and activate immunity has been attributed to its capacity to phosphorylate and inactivate the E3 ligase domain of AvrPtoB. Here we report that Pto can bind two distinct domains of AvrPtoB. Pto bound to the domain near the E3 ligase is degraded, whereas the distally bound Pto escapes ubiquitination. Furthermore, a kinase-inactive variant of Pto is fully capable of activating immunity in response to AvrPtoB, showing that proximity to the E3 ligase domain and not effector phosphorylation determines Pto recalcitrance to degradation. Our study provides further insight into the mechanism evolved by tomato to counteract a pathogenicity determinant of a bacterial pathogen, allowing it to activate an effective immune response.

## Introduction

In the perpetual evolutionary arms race between hosts and pathogens, plants evolved two layers of inducible defense to protect themselves from infection [1]. The first layer is now commonly referred to as pattern-triggered immunity (PTI). At its core are cell surface host receptors that detect common, highly conserved molecular features of microbes, referred to as microbe-associated molecular patterns. These receptors activate a relatively mild but effective defense response that includes the release of reactive oxygen species, changes in gene expression, and cell wall fortification [2], [3]. While this response is sufficient to prevent many potentially pathogenic microbes from successfully establishing an infection, adapted pathogens have evolved large arsenals of ‘effector’ proteins [4]–[6]. These effectors are typically delivered into the plant cell cytoplasm, where they interfere with immune signaling to subvert PTI [7]. The second plant immune response addresses this threat by monitoring for the presence of pathogen effectors and is consequently referred to as effector-triggered immunity (ETI) [8]–[10]. Because detection of an effector indicates the presence of a highly adapted and potentially devastating pathogen, the immune reaction is stronger and often includes localized cell death, referred to as the hypersensitive response [11]–[13]. Collectively, the ETI responses prevent the successful colonization by biotrophic or hemibiotrophic pathogens.

The interaction of *Pseudomonas syringae* pv. *tomato* DC3000 (*Pst*) with tomato is one of the best understood plant pathosystems [11], [14]. *Pst* delivers ∼28 effectors into the host cytoplasm via its type III secretion system in order to suppress PTI [4]. In response, certain wild relatives of tomato have evolved the capacity to recognize two of these effectors, AvrPto and AvrPtoB [15]. These proteins are unrelated, but both can be bound by the host Pto kinase which then acts with an NB-LRR protein, Prf, to trigger ETI [16]–[21]. *Pto* is a member of a small gene family in tomato and another family member, *Fen*, encodes a protein that is also able to bind AvrPtoB [22], [23]. However, the C-terminus of AvrPtoB contains a U-box type E3 ubiquitin ligase domain that marks Fen for degradation by the proteasome, thereby undermining Fen-mediated immunity [22], [24]–[27]. If the E3 ligase domain is incapacitated, Fen is able to activate ETI in conjunction with Prf [22].

Although Pto and Fen have 87% amino acid similarity, Fen is efficiently ubiquitinated by the AvrPtoB E3 ligase and degraded, whereas Pto is recalcitrant to ubiquitination and capable of activating ETI in response to binding AvrPtoB [22]. Understanding the molecular basis for this difference could shed light on an evolutionary step in the tomato-*Pst* interaction and also reveal an interesting mechanism underlying ETI. It has been reported recently that Pto is protected from AvrPtoB-mediated degradation because it has a significantly higher kinase activity than Fen [28]. This increased kinase activity was proposed to enable Pto to more efficiently phosphorylate AvrPtoB specifically at threonine-450 and this modification was reported to inactivate the AvrPtoB E3 ligase domain, allowing Pto to escape degradation [28].

A second important difference between Pto and Fen relates to the subdomains of AvrPtoB that they bind. A set of truncated versions of the effector was tested for interaction with both protein kinases, and only Pto was found to interact with an N-terminal fragment contained within amino acids 1–307 [22], [27], [29]. This region of AvrPtoB is also known to bind another tomato kinase, Bti9, thereby interfering with its role in PTI [30]. The structure of Pto in complex with this N-terminal binding site on AvrPtoB has been resolved by x-ray crystallography [17]. In contrast, Fen cannot interact with AvrPtoB_1–307_, but requires a longer fragment spanning amino acids 1-387 for interaction [22]. While Fen has so far eluded structural analysis, the additional C-terminal region of the effector necessary for interaction with Fen contains the binding site for the PTI co-receptor kinase BAK1, an important virulence target of AvrPtoB [25], [31].

A current model postulates that the AvrPtoB BAK1-interacting domain (BID) originally evolved as a virulence determinant to bind and suppress host kinases involved in PTI [15], [22]. Fen evolved as a decoy of these kinases, allowing it to interact with the BID (for clarity, here we will refer to this domain as the Fen-interacting domain, FID) and activate ETI [15]. The FID region of *avrPtoB* underwent an intragenic duplication creating a structurally similar N-terminal domain that diverged to target additional host protein kinases involved in PTI [25], [30]. The AvrPtoB E3 ligase domain was then acquired which effectively defeated *Fen* leading to disease susceptibility. However, multiple duplications of the *Fen* gene occurred in tomato and the protein encoded by one of the resulting genes, *Pto*, gained the ability to bind the N-terminal domain of AvrPtoB (i.e., the Pto-interacting domain, PID) and resist ubiquitination by the AvrPtoB E3 ligase. This stand-off appears to be the current stage in this plant-pathogen co-evolutionary arms race.

Here we investigate how Pto binds AvrPtoB and the mechanism by which this host kinase is able to resist ubiquitination by the AvrPtoB E3 ligase and activate the plant immune response.

## Results

### Pto binds two distinct domains of AvrPtoB, whereas Fen binds only an E3 ligase-proximal domain

**Figure 1:**
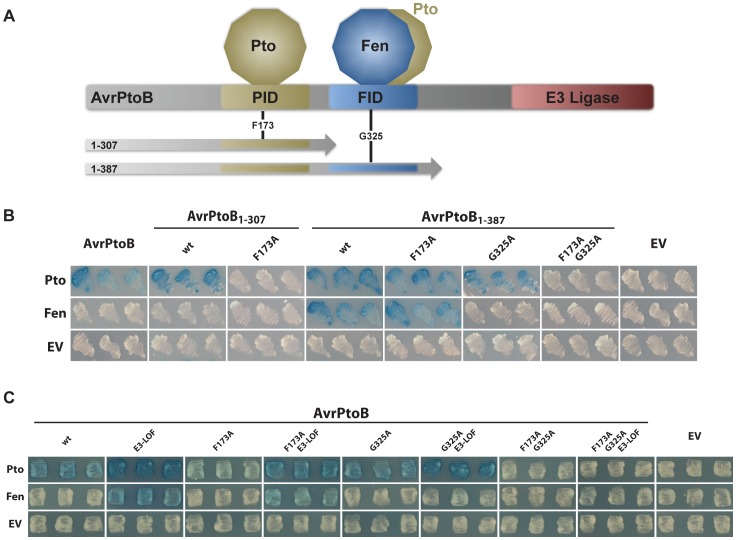
Pto, but not Fen, binds the PID of AvrPtoB and is recalcitrant to E3 ligase-mediated degradation. (A) Schematic of AvrPtoB functional domains and truncations used in this and previous publications [24]. PID, Pto-interacting domain [29]; FID, Fen-interacting domain (also known as the Bak1-interacting domain [25]). Position of amino acid substitutions, F173A and G325A, are shown. (B) Yeast two-hybrid analyses testing the interaction of tomato Pto and Fen kinases (in the bait vector) with different functional domains in AvrPtoB using truncated forms of the effector (in the prey vector). Blue patches indicate a positive interaction. Pto interacted with both the PID and the FID, whereas Fen exclusively bound the FID. wt, wild-type AvrPtoB in the truncation indicated; EV, empty vector. (C) Yeast two-hybrid analyses of the binding properties of tomato Pto and Fen kinases (in the bait vector) towards full-length AvrPtoB protein (in the prey vector). Blue patches indicate a positive interaction. Pto bound to the FID, proximal to the E3 ubiquitin ligase domain, was degraded in an E3-ligase dependent manner, similar to Fen. E3-LOF, AvrPtoB(F479A/F525A/P533A) has substitutions that abolish binding of the E2 conjugating enzyme and lacks E3 ligase activity [26]; EV, empty vector.

The closely related kinases, Pto and Fen, from *Solanum pimpinellifolium* differ in their interactions with AvrPtoB [22], [29]. Pto interacts with the Pto-interacting domain (PID) contained within amino acids 1–307 of the effector (AvrPtoB_1–307_), whereas Fen interacts exclusively with the Fen-interacting domain (FID) spanning amino acids 307–387, present in the fragment AvrPtoB_1–387_ (**Fig. 1A**). To further examine these interactions, we subjected a number of AvrPtoB variants, with point mutations in the PID and FID domains, to yeast two-hybrid analyses with Fen and Pto (**Fig. 1B**). As reported previously, Pto interacted with AvrPtoB_1–307_, but was unable to interact with AvrPtoB_1–307_(F173A) [32]. Pto also interacted with AvrPtoB_1–387_, because this fragment contains the 1–307 region, but Fen interacted only with AvrPtoB_1–387_. Surprisingly, we found that Pto also interacted strongly with the AvrPtoB_1–387_(F173A) protein in which the PID has been inactivated. This indicated that Pto binds two distinct domains of AvrPtoB, the PID and FID, whereas Fen interacts only with the FID. To corroborate this finding, we included an AvrPtoB variant, AvrPtoB_1–387_(G325A) that is unable to interact with Fen. We found that the G325A substitution abolished the interaction of both Fen (no interaction of Fen with AvrPtoB_1–387_(G325A)) and Pto (no interaction of Pto with AvrPtoB_1–387_(F173A/G325A)) with the FID, but had no impact on the binding of Pto with the PID (strong interaction of Pto with AvrPtoB_1–387_(G325A)). These findings indicate that Fen interaction with AvrPtoB is limited to the FID, whereas Pto has the ability to bind both the FID and the PID.

### Pto that binds to the E3 ligase-proximal FID is susceptible to degradation similar to Fen

To gain further insight into the interactions of Pto and Fen with AvrPtoB, we generated all combinations of amino acid substitutions in the FID and PID in full-length constructs that had an inactive E3 ligase (E3-LOF, having substitutions in the three E2-conjugating enzyme binding sites: F479A/F525A/P533A) (**Fig. 1C**). In the context of this full-length effector with an inactive E3 ligase, both Pto and Fen showed the same binding capabilities towards AvrPtoB as towards the truncated forms. Specifically, Pto could bind to both the PID and the FID, but Fen bound only to the FID. The presence of E3 ligase activity in the AvrPtoB C-terminus masked the interaction with Fen by marking the kinase for degradation, as described previously [22], [26]. Interestingly, if Pto was forced to bind exclusively to the FID by inclusion of the F173A substitution in the PID, this interaction was also masked by the E3 ligase activity, similar to Fen binding the same domain. These observations suggest that Pto is not intrinsically resistant to AvrPtoB-mediated degradation, but rather the proximity of its binding relative to the effector E3 ligase domain impacts whether or not Pto is ubiquitinated. We refer to this as the ‘proximity’ hypothesis.

### Positioning of the PID directly adjacent to the E3 ligase domain renders Pto susceptible to AvrPtoB-mediated degradation

**Figure 2:**
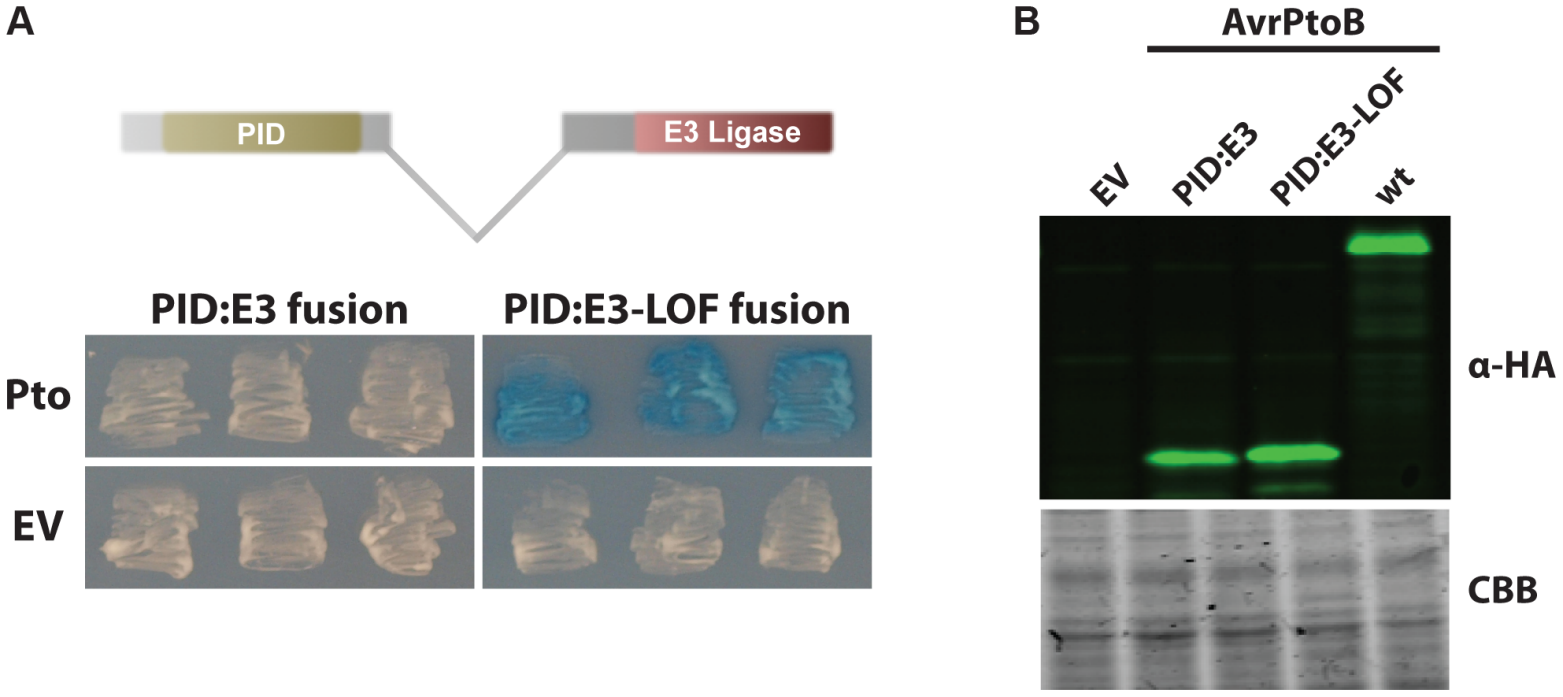
AvrPtoB mediates Pto degradation if the kinase is positioned closer to the AvrPtoB E3 ligase. (A) The AvrPtoB PID was fused to the E3 ligase domain, cloned into the prey vector (see Methods for details) and tested for its interaction with Pto (in the bait vector). Blue patches indicate a positive interaction. Pto was degraded if it was in closer proximity to the E3 ligase domain as shown by the white patches. E3-LOF, AvrPtoB(F479A/F525A/P533A) lacks E3 ligase activity; EV, empty vector. (B) Expression levels of both fusion proteins and wild-type AvrPtoB are similar. Yeast cells grown in inductive medium were harvested, normalized by OD_600_, boiled in Laemmli buffer and proteins resolved by Western blotting. Fusion proteins were detected using ant-HA primary (clone 3F10, Roche, Indianapolis, IN, USA) and anti-rat-800 secondary (IRDye 800CW, LI-COR, Lincoln, NE, USA) antibodies and visualized using an Odyssey Scanner (LI-COR). CBB, Coomassie brilliant blue staining.

We tested the proximity hypothesis by generating a synthetic protein in which the PID (amino acids 121–200) was fused directly to the E3 ligase domain (amino acids 388–533) (**Fig. 2**). This PID fusion protein interacted strongly with Pto when the E3 ligase domain was inactivated. Significantly, this interaction was abrogated in the presence of an active E3 ligase domain, similar to what occurs when Fen or Pto bind the FID, indicating Pto is degraded when it binds a domain proximal to the E3 ligase domain. A Western blot showed that the PID fusion proteins were expressed as well as wild-type AvrPtoB (**Fig. 2B**) and the positive result of the interaction between Pto and PID:E3-LOF indicates that the kinase is expressed.

### Pto binding of the FID activates ETI in tomato and *Nicotiana benthamiana* leaves

**Figure 3:**
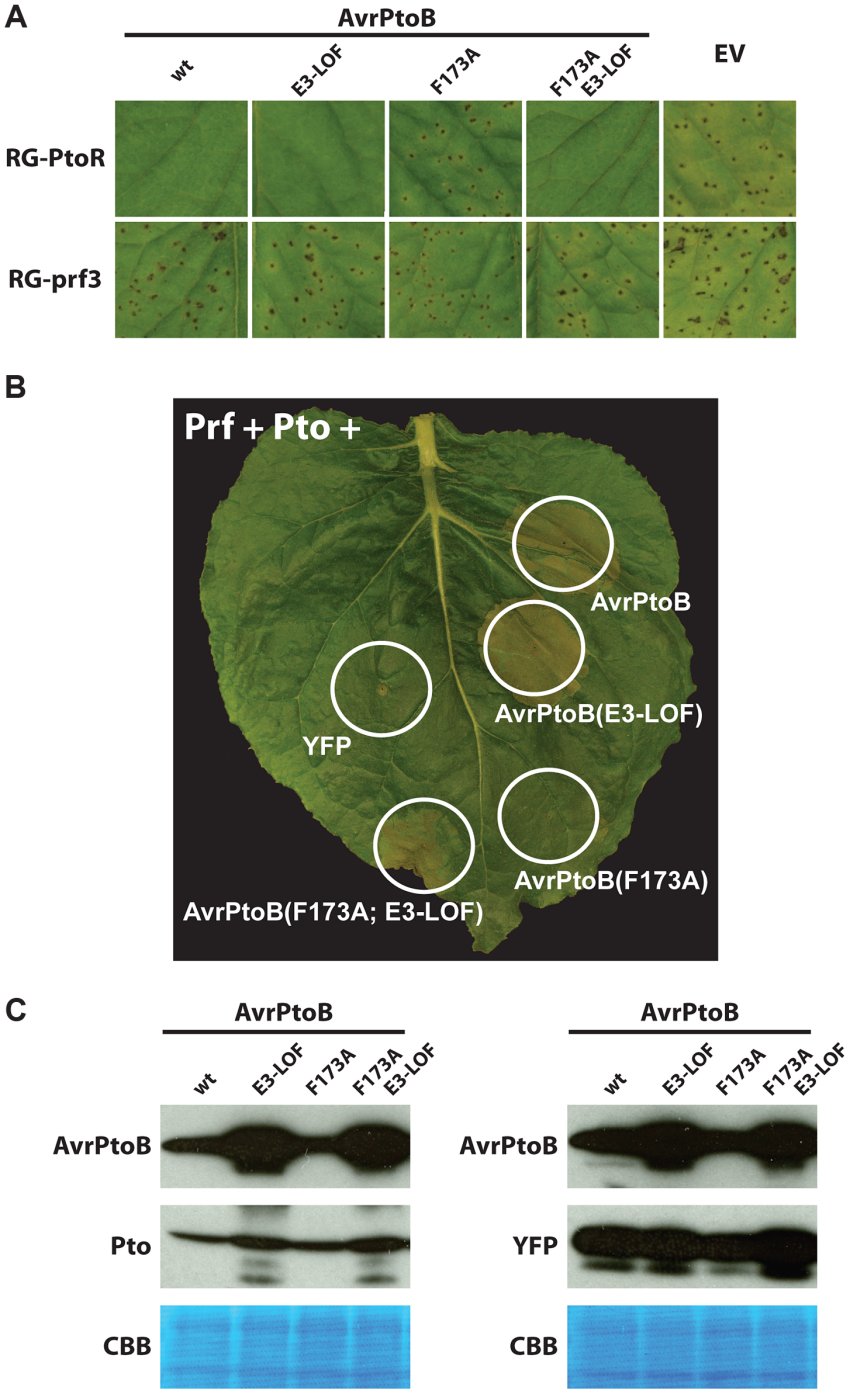
Pto binds to the FID in plant cells and is degraded by activity of the AvrPtoB E3 ligase. (A) The *P. syringae* pv. *tomato* strain DC3000Δ*avrPto*Δ*avrPtoB* was transformed with wild-type (wt) AvrPtoB or AvrPtoB variants under control of an *Pst hrp* promoter and infiltrated into leaves of Rio Grande (RG) tomatoes. Disease phenotypes were consistent with the observations in yeast two-hybrid analyses. RG-PtoR (*Pto/Pto, Prf/Prf)*; RG-prf3 (*Pto/Pto, prf3/prf3)*. RG-prf3 has a deletion in *Prf* that inactivates the Pto/Prf pathway. (B) Tomato Pto and Prf were co-expressed with different variants of AvrPtoB in a leaf of *Nicotiana benthamiana* by using *Agrobacterium-*mediated transient transformation. Pto activated ETI, as manifested by cell death with AvrPtoB(F173A)-E3-LOF, upon binding the FID but this response was repressed by the E3 ligase domain with no cell death visible with AvrPtoB(F173A), similar to Fen binding of the same domain. E3-LOF, AvrPtoB(F479A/F525A/P533A) lacks E3 ligase activity; YFP, yellow fluorescent protein control. (C) Abundance of Pto is decreased when it is co-expressed with AvrPtoB variants having an active E3 ligase domain. Using *Agrobacterium-*mediated transient transformation, tomato Pto was co-expressed with different variants of AvrPtoB in a leaf of a *Prf*-silenced *Nicotiana benthamiana* plant (to avoid the possible effect of cell death on protein abundance). Leaf samples were harvest 48 hrs after agroinfiltration and proteins were isolated for Western blot analysis. Similar experiments were done with yellow fluorescent protein (YFP) as a loading control. Proteins were detected using an anti-c-Myc-HRP antibody targeted to the epitope tag fused to each protein. Exposure was 1 min for AvrPtoB and Pto, and 10 seconds for YFP. CBB, Coomassie brilliant blue staining.

We transformed *Pst* DC3000Δ*avrPto*Δ*avrPtoB* with the different variants of *avrPtoB* under control of a *Pst hrp*-inducible promoter and used these strains to infiltrate leaves of tomato plants that are either resistant (expressing *Pto* and *Prf*; Rio Grande-PtoR, RG-PtoR) or susceptible (carrying a mutation in *Prf*; Rio Grande-prf3, RG-prf3) (**Fig. 3A**). As observed previously, AvrPtoB(F173A) was unable to elicit Pto-mediated immunity [32]. However, in agreement with our yeast data, inactivation of the E3 ligase domain restored the immune response. A *Fen* knockout tomato line is not available thus making it impossible to test whether this reconstituted immune response is due to Pto or Fen (or both) binding to the FID. A tomato line lacking *Prf* (RG-prf3) and therefore deficient in both Fen- and Pto-mediated immunity was susceptible to all strains as expected (**Fig 3A**).

To address the unavailability of tomato line lacking *Fen*, we employed *Agrobacterium*-mediated transient expression in *Nicotiana benthamiana*, a system that has been successfully used to investigate Pto- and Fen-mediated immunity [24], [27], [33]. It has been reported that one or more Pto family members in *N. benthamiana* recognize AvrPtoB_1–387_ and not AvrPtoB_1-307_ and can be degraded by the E3 ligase activity in AvrPtoB, analogous to degradation of Fen in tomato (referred to as the ‘Rsb’ phenotype; [22], [24]). However, when we tested new constructs expressing these truncations with C-terminal tags, we found unexpectedly that AvrPtoB_1–307_ was sufficient to activate Prf-dependent cell death in *N. benthamiana* (**Fig. S1**). Furthermore, the F173A mutation was sufficient to completely abolish recognition of E3 ligase-inactive AvrPtoB in *N. benthamiana*. We believe the earlier observations that *N. benthamiana* recognizes only AvrPtoB_1–387_ were due to low expression levels of the original AvrPtoB_1–307_ construct, which was not epitope-not tagged and for which expression levels were not tested [24].

The finding that the F173A substitution abolished the ability of AvrPtoB to elicit ETI-associated cell death in *N. benthamiana* gave us the opportunity to test whether Pto binding to the FID can activate ETI and, if so, whether this response can be suppressed by the AvrPtoB E3 ligase domain (**Fig. 3B**). It has previously been shown that tomato Pto can recognize full-length AvrPtoB in *N. benthamiana* if it is co-expressed with the tomato *Prf* gene [33]. In agreement with our yeast results, co-expression of *Pto* and *Prf* with *avrPtoB*(F173A/E3-LOF) caused ETI-associated cell death in *N. benthamiana* and this response was suppressed by the presence of a functional E3 ligase in AvrPtoB(F173A). A Western blot indicated that, as expected, Pto abundance was decreased in the presence of wild type AvrPtoB and AvrPtoB(F173A), but unaffected in the presence of the E3-LOF variants of AvrPtoB (**Fig. 3C**). Note that the E3-LOF variants of AvrPtoB are expressed at a higher level that the E3 ligase active versions (probably because AvrPtoB autoubiquitinates and is degraded). Nevertheless, even with this increased expression, AvrPtoB-E3-LOF variants do not cause a decrease in the abundance of Pto. These results demonstrate not only that Pto can bind the FID and trigger ETI, but also that the E3 ligase domain can inhibit this Pto-mediated response just as it inhibits cell death activated by Fen binding to the FID.

### Pto recalcitrance to degradation does not depend on phosphorylation of T450 in AvrPtoB

**Figure 4:**
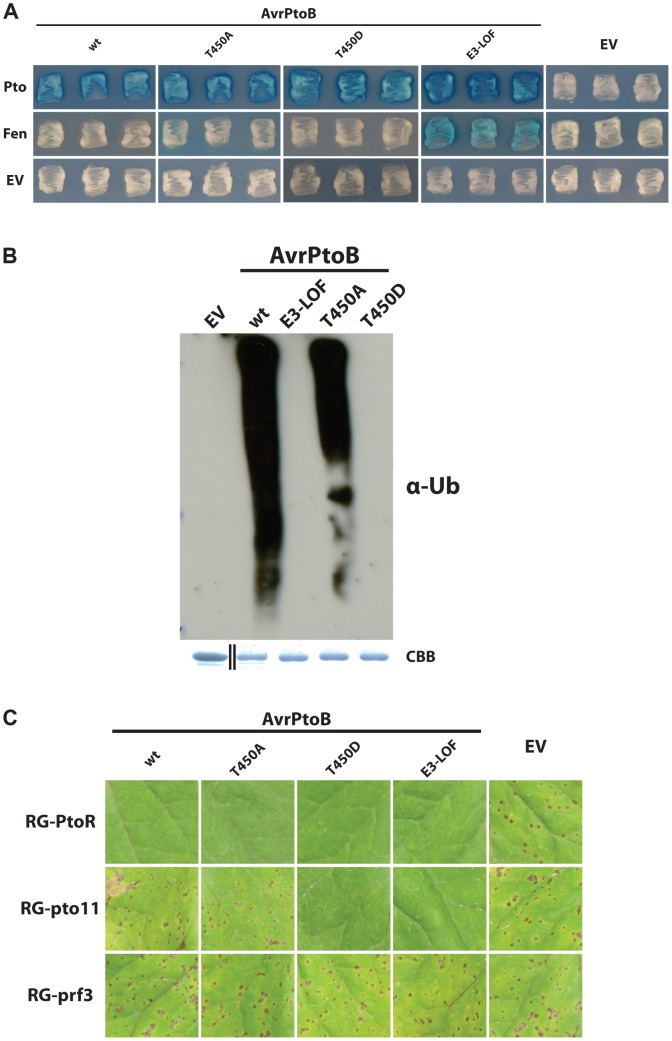
Pto-mediated phosphorylation of T450 in AvrPtoB does not impact Pto recalcitrance to E3 ligase-mediated degradation. (A) Yeast two-hybrid analyses of the interaction of AvrPtoB(T450A) and AvrPtoB(T450D) with Pto and Fen. AvrPtoB or the variants shown (in the prey vector) were tested for their interaction with Pto and Fen. Blue patches indicate a positive interaction. Substitution of T450 in AvrPtoB by the non-phosphorylatable residue alanine had no impact on either the capability of the effector to degrade Fen or the recalcitrance of Pto to this degradation. AvrPtoB(T450D) interacted with Pto as expected based on results in (B) and (C) below. The reason for the lack of Fen interaction with AvrPtoB(T450D) is unknown as this interaction would be expected to occur based on (B) and evidently does occur in the plant-pathogen interaction as shown in (C). E3-LOF, AvrPtoB(F479A/F525A/P533A) lacks E3 ligase activity; EV, empty vector. (B) *In vitro* ubiquitination assay to determine ubiquitin ligase activities of different variants of AvrPtoB. GST-fusions of the effector were purified from *E. coli* and subjected to an *in vitro* ubiquitination assay. After Western blotting, poly-ubiquitin chains were detected using a monoclonal anti-ubiquitin antibody (α-Ub). In contrast to previous reports, AvrPtoB(T450A) retained significant E3 ubiquitin ligase activity. CBB, Coomassie Brilliant Blue. Black divider line in loading control panel demarcates a copy/paste border as GST has a much smaller mass than the fusion proteins. (C) The *P. syringae* pv. *tomato* strain DC3000Δ*avrPto*Δ*avrPtoB* was transformed with wild-type (wt) or variants of AvrPtoB under control of a *Pst hrp* promoter and infiltrated into Rio Grande (RG) tomatoes. AvrPtoB(T450D) was recognized both by Pto (no disease in RG-PtoR) and Fen (no disease in the absence of Pto in RG-pto11) and mimics the E3-LOF version of the effector. In contrast, AvrPtoB(T450A) behaved identical to wild-type AvrPtoB. Fen-mediated immunity is suppressed, but Pto can still resist degradation and confer immunity, demonstrating that phosphorylation of T450 in AvrPtoB is not required for Pto to resist degradation *in vivo* thus confirming the *in vitro* finding that AvrPtoB(T450A) has E3 ligase activity. RG-PtoR, *Pto/Pto, Prf/Prf*; RG-pto11, *pto11/pto11, Prf/Prf*; RG-prf3, *Pto/Pto, prf3/prf3*. RG-pto11 has a point mutation that produces a non-functional Pto protein. RG-prf3 has a deletion in *Prf*.

It has been reported that Pto has greater kinase activity than Fen, allowing it to more effectively phosphorylate threonine-450 (T450) in AvrPtoB [28]. This phosphorylation was reported to inactivate the E3 ligase of the effector, explaining how Pto, but not Fen, resists degradation and activates an immune response. Our results suggest that the site at which Fen and Pto bind AvrPtoB relative to its E3 ligase domain has an impact on the fate of the kinase and consequently on disease resistance. These observations could support a model in which the ability of Pto to bind the PID in addition to the FID allows it to more effectively phosphorylate T450 of AvrPtoB. To test this hypothesis, we generated an AvrPtoB protein with a T450A substitution and tested its interaction with Pto in yeast (**Fig. 4A**). We expected one of two outcomes: 1) the T450A mutation disrupts E3 ligase activity similar to the published T450D substitution [28], allowing AvrPtoB(T450A) to interact with both Fen and Pto; or 2) the T450A substitution has no impact on E3 ligase activity but prevents Pto from phosphorylating and inactivating AvrPtoB, leading to degradation of Pto and evasion of Pto-mediated immunity *in vivo*. However, the outcome of this experiment did not agree with either expectation. Instead, AvrPtoB(T450A) was indistinguishable from wild-type AvrPtoB in that it interacted with Pto, but masked its interaction with Fen (**Fig. 4A**). This was puzzling as AvrPtoB(T450A) reportedly has no E3 ligase activity *in vitro* [28].

To resolve this apparent contradiction, we repeated the experiment from Ntoukakis et al. [28] by purifying AvrPtoB(T450A) from *E. coli* and subjecting it to an *in vitro* E3 ligase assay (**Fig. 4B**). Consistent with our observation that AvrPtoB(T450A) can interact with Pto and cause degradation of Fen, this effector variant had only slightly reduced E3 ligase activity compared to wild-type AvrPtoB, whereas AvrPtoB(T450D) completely lacked E3 ligase activity.

This raised the question whether the slight reduction in E3 ligase activity caused by the T450A substitution is biologically significant or, as our results in yeast indicate, has no effect on the interplay between AvrPtoB and Pto. To make this distinction and stay as close to the *in vivo* situation as possible, we transformed *Pst* DC3000Δ*avrPto*Δ*avrPtoB* with different variants of *avrPtoB* under the control of a *hrp*-inducible promoter. The resulting strains were used to inoculate tomato leaves and disease or resistance outcomes were documented (**Fig. 4C**). RG-prf3 plants (lacking a functional Prf) served as a positive control for virulence; all of the strains were able to cause disease in this tomato line. RG-PtoR plants (expressing Pto and Prf) served to demonstrate the delivery of the AvrPtoB variants into the plant cell. In contrast to the observations with RG-prf3, none of the bacterial strains expressing any of the AvrPtoB variants, including AvrPtoB(T450A), caused disease in RG-PtoR plants. This result demonstrates that each of the AvrPtoB variants is successfully delivered into the plant cell cytoplasm, where Pto binding initiates ETI.

We next tested the bacterial strains on RG-pto11, a tomato line that has a nonfunctional *Pto* gene, rendering them susceptible to *Pst* expressing wild-type AvrPtoB (**Fig. 4C**). RG-pto11 plants do have a functional Fen, however, and are resistant to *Pst* with forms of AvrPtoB lacking E3 ligase activity [22], [24]. As expected, RG-pto11 tomatoes were susceptible to DC3000Δ*avrPto*Δ*avrPtoB* lacking AvrPtoB or expressing wild-type AvrPtoB, but resistant to strains with the E3 ligase-inactive variant AvrPtoB(E3-LOF). In agreement with both our yeast two-hybrid and E3 ligase assays as well as previously published data [28], RG-pto11 plants were also able to recognize AvrPtoB(T450D). However, RG-pto11 plants were unable to recognize AvrPtoB(T450A), indicating this effector variant is still able to suppress Fen-mediated ETI, which requires an active E3-ligase domain. This demonstrated not only that AvrPtoB(T450A) has E3 ligase activity, but also that the slight reduction in E3 ligase activity observed in the *in vitro* assays does not impact the plant-pathogen interaction. This result is consistent with our yeast two-hybrid analyses (**Fig. 4A**). Taken together with our observation that RG-PtoR plants are able to recognize AvrPtoB(T450A), this demonstrates that Pto phosphorylation of T450 in AvrPtoB is dispensable for Pto-mediated ETI triggered by AvrPtoB.

### The relative *in vitro* kinase activities of Fen and Pto are dependent on buffer conditions

Ntoukakis et al. [28] reported a much reduced kinase activity for Fen relative to Pto and concluded this difference was a key factor in the ability of Pto, but not Fen, to phosphorylate T450 and thereby inactive the E3 ligase of AvrPtoB. These findings differ from earlier papers that described Pto and Fen as active kinases, with similar activities *in vitro* [34]–[37]. There are several differences in the protocols used in each case that might explain these discrepancies. In earlier assays from our laboratory, the kinases were expressed and purified as N-terminal maltose-binding protein (MBP)-fusions and the kinase assays were performed at pH 7.0 with 10 mM MnCl_2_ [34]–[37]. Ntoukakis et al. [28], however, purified C-terminally His-fusions and performed their assays at pH 7.5 with 10 mM MgCl_2_ and 1 mM MnCl_2_.

**Figure 5:**
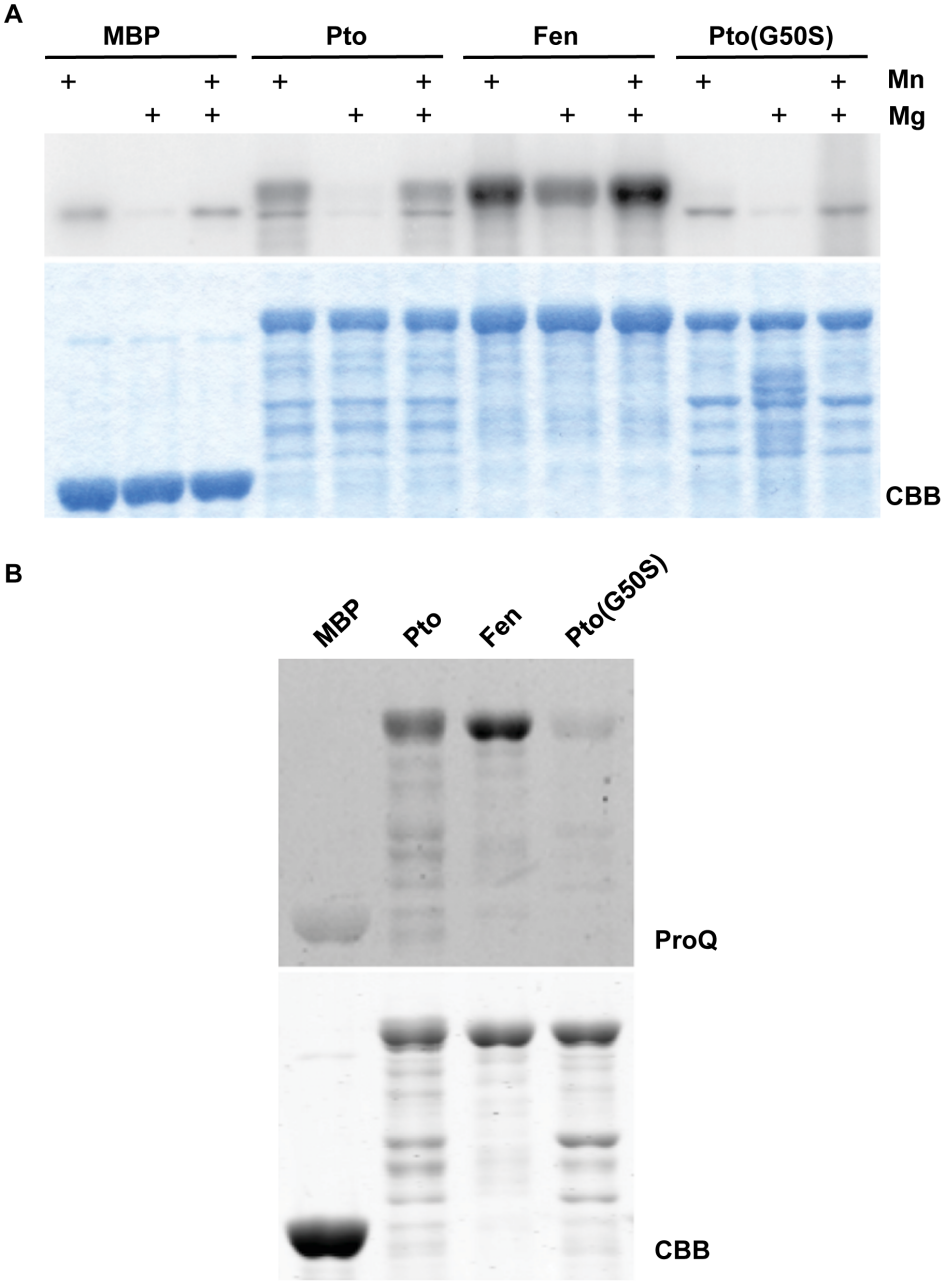
Fen has higher kinase activity than Pto and Pto(G50S) has little or no kinase activity. (A) *In vitro* kinase assay for tomato Pto, Fen, and Pto(G50S) at pH6.8. At this pH, no brown discoloration was visible upon addition of 10 mM MnCl_2_, indicating a better availability of Mn^2+^. Kinase buffers were supplemented with 10 mM MnCl_2_, 10 mM MgCl_2_ or 10 mM of each. Under these conditions, Fen showed a higher kinase activity than Pto and Pto(G50S) had little or no kinase activity. Coomassie Brilliant Blue (CBB) staining showed similar amounts of the kinases were present. (B) Phosphoprotein-specific ProQ staining of Pto, Fen and Pto(G50S) to assess their phosphorylation status in bacteria. Pto and Fen were expressed in *E. coli*, pulled down using MBP-agarose, resolved by SDS-PAGE and subjected to ProQ staining. Stronger staining of Fen indicates a higher autophosphorylation activity *in situ*. Pto(G50S) had little or no kinase activity in this *in vivo* assay. Coomassie Brilliant Blue (CBB) staining showed similar amounts of the kinases were present.

We reasoned that the different metal ions present in the assays were a likely explanation for the differences in kinase activities. Therefore we tested kinase activities with 10 mM MnCl_2_ and 10 mM MgCl_2_ at pH 7.5 and did observe higher kinase activity for Pto in the presence of 10 mM MnCl_2_ (**Fig. S2A**). However, we observed a brown discoloration of the kinase buffer at pH 7.5 in the presence of 10 mM MnCl_2_ (data not shown). We hypothesized this was due to a pH-dependent complex formation of Mn with Tris-HCl in solution. Indeed, such a pH-dependent complex formation has been described in the literature [38]. Further investigation showed that the observed discoloration increased with increasing pH and was dramatically enhanced upon addition of 1 mM DTT, as was used in all the kinase assays mentioned above, to the Tris buffers above pH 7.0 (**Fig. S2B**). This complex formation, together with the ten-fold lower MnCl_2_ concentration used by Ntoukakis et al. [28], could lead to a situation where the availability of Mn^2+^ is a limiting factor. To test whether the different kinase activities of Fen and Pto are due to limited availability of Mn at pH 7.5, we performed kinase assays with both proteins at pH 6.8. At pH 6.8 kinase activity of Fen was higher than that of Pto and this increased activity was dependent on the presence of Mn in the buffer (**Fig. 5A**).

The finding that the relative kinase activities of Fen and Pto are dependent on buffer pH and ion availability represents a general problem of *in vitro* experimentation. The chosen buffer conditions are to a certain degree arbitrary, and it is difficult to predict or replicate the exact *in vivo* conditions in the relevant subcellular locale. One way to address this limitation is to assess autophosphorylation activity of the kinases inside a living cell. Taylor et al. [39] recently published that many plant kinases, when expressed in *E. coli*, retain kinase activity and readily autophosphorylate. Furthermore, the level of this phosphorylation, as determined by using the phosphoamino acid stain Pro-Q Diamond, is indicative of the activity of the kinase under investigation [39]. We purified Fen and Pto from *E. coli* and assessed their phosphorylation status *in vivo* using Pro-Q staining (**Fig. 5B**). These experiments revealed that Fen autophosphorylation was approximately two- to four-fold higher than that of Pto. Together with our *in vitro* kinase assays in the presence of Mn, this indicates that under certain conditions Fen is a moderately more active kinase than Pto.

### A Pto variant that lacks kinase activity still activates ETI in response to AvrPtoB

Our results suggest that Pto is not necessarily a more active kinase than Fen and we demonstrated that Pto phosphorylation of T450 is not required for its recalcitrance to AvrPtoB-mediated degradation. However, it is conceivable that in the absence of a phosphorylatable residue at position 450 and under the right conditions, Pto could phosphorylate neighboring amino acids which inactivate the E3 ligase domain. In fact, AvrPtoB has four serine residues within a distance of ten amino acids upstream or downstream of T450 that could serve as phospho-acceptors. We reasoned that the best way to exclude a role for Pto kinase activity in recalcitrance to AvrPtoB-mediated degradation would be to use a form of Pto that is kinase inactive, or at least less active than Fen under all conditions, but still capable of activating ETI. One Pto variant, Pto(G50S), was reported previously to retain the ability to activate ETI in response to AvrPto [33], [40]. G50 is an invariant residue in kinase subdomain I that plays a key role in binding ATP and its substitution would be expected to diminish or abolish kinase activity [41]. Indeed, when we purified Pto(G50S) from *E. coli* and subjected it to an *in vitro* assay we found that it had little or no kinase activity (**Fig. 5A**), a result confirmed by Pro-Q staining (**Fig. 5B**).

**Figure 6:**
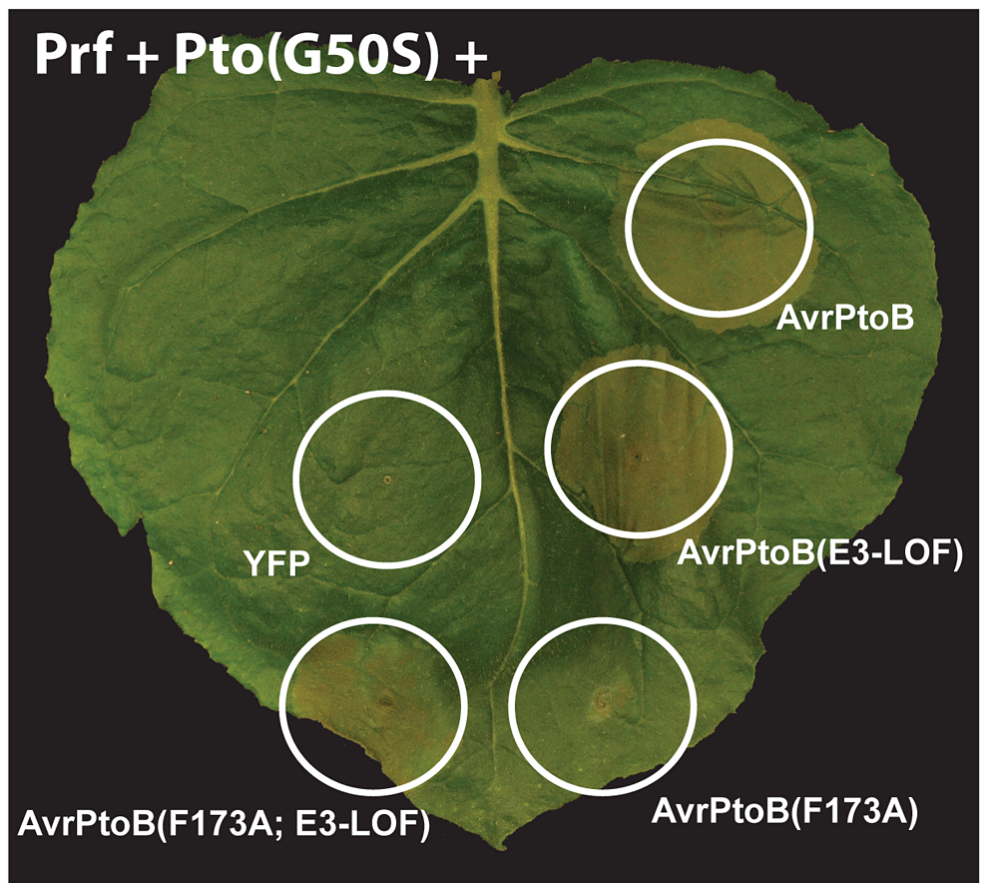
Pto and Fen kinase activities are dispensable for activation of AvrPtoB-elicited ETI. The Pto(G50S) variant which has little or no kinase activity (see Fig. 5BC) and tomato Prf were co-expressed with different variants of AvrPtoB in a leaf of *Nicotiana benthamiana* by *Agrobacterium-*mediated transient transformation. The response of Pto(G50S) to the AvrPtoB variants was indistinguishable from wild-type Pto in that it caused ETI-associated cell death upon binding the FID (cell deaths with AvrPtoB(F173A; E3-LOF)), but this response was repressed by the E3 ligase domain (no cell death by AvrPtoB(F173A)).

The transgenic tomato line expressing Pto(G50S) [40] is no longer available, so we utilized transient expression in *N. benthamiana* to test the ability of Pto(G50S) to activate ETI in response to AvrPtoB (**Fig. 6**). When co-expressed with tomato *Prf*, Pto(G50S) triggered ETI-associated cell death in response to AvrPtoB. In addition, as shown above for wild-type Pto (**Fig. 3B**), co-expression of *Pto(G50S)* with *avrPtoB(F173A)* did not activate ETI, but co-expression with an *avrPtoB(F173A/E3-LOF)* did. We conclude that with respect to its ability to recognize AvrPtoB and activate ETI, the kinase inactive Pto(G50S) is indistinguishable from wild-type Pto, demonstrating that Pto kinase activity is not required for Pto recalcitrance to AvrPtoB-mediated degradation.

## Discussion

Testing variant forms of AvrPtoB for their interaction with Pto and Fen in a yeast two-hybrid system, revealed that Pto is capable of binding two distinct domains in the effector, both the N-terminal PID and the E3-ligase proximal FID, whereas Fen binds exclusively to the FID (**Fig. S3**). Furthermore, Pto that binds the FID is subjected to AvrPtoB-mediated degradation just as is Fen, showing that Pto is not intrinsically resistant to degradation. We also tested a construct in which the PID was fused directly to the E3 ligase domain and observed that Pto bound to this E3 ligase proximal PID was degraded. We initially interpreted these results as an indication that the position at which the kinases bind AvrPtoB relative to the E3 ligase domain might be the underlying cause for their apparent differential ability to phosphorylate T450 in AvrPtoB. We hypothesized that the unique ability of Pto to bind the more N-terminal PID allows Pto to efficiently phosphorylate AvrPtoB and thereby repress its E3 ligase activity. In contrast, Fen, which can only bind the more C-terminal FID, would not be in the optimal position and/or orientation to phosphorylate T450 and consequently be subject to ubiquitination and degradation.

When we tested the ability of Pto to activate ETI in response to AvrPtoB, we found that in the absence of E3 ligase activity Pto can trigger ETI upon binding either domain. However, only the Pto that binds the PID can activate ETI in the presence of an active AvrPtoB E3 ligase domain. Pto that binds the FID is targeted for degradation by the E3 ligase similar to Fen. These results are consistent with our yeast two-hybrid analyses and confirm that the binding sites of Pto and Fen on AvrPtoB determine the outcome. The presumed later evolution of the ability of Pto to bind the PID in addition to the FID allows it to escape AvrPtoB-mediated degradation and ultimately to trigger ETI.

To investigate the role of T450 phosphorylation in this process, we generated a version of AvrPtoB that can no longer be phosphorylated at this residue. Our results show that AvrPtoB(T450A) causes degradation of Fen in yeast and is capable of efficiently suppressing Fen-mediated ETI in tomato, properties that are identical to wild-type AvrPtoB. These observations were unexpected, as AvrPtoB(T450A) had previously been reported to lack E3 ligase activity *in vitro* [28]. When we repeated those experiments, we found that AvrPtoB(T450A) had only slightly reduced E3 ligase activity compared to wild-type AvrPtoB. The observation that AvrPtoB(T450A) still suppresses Fen-mediated ETI in tomato shows that this minor reduction is biologically insignificant. These results demonstrate that phosphorylation of T450, if that is what happens during the infection process, is not necessary for Pto-mediated ETI. The possibility remained that in the absence of a phosphorylatable residue at position 450, Pto might phosphorylate neighboring serine residues and thus interfere with E3 ligase activity.

The results described above demonstrate that the different binding characteristics of Pto and Fen underlie their differential vulnerability to degradation by AvrPtoB. They do not, however, exclude the possibility that a higher kinase activity of Pto suppresses AvrPtoB E3 ligase activity, assuming that other suitable phospho-acceptor residues exist in addition to T450. Unfortunately, there are inconsistent reports regarding the kinase activities of Pto and Fen. In some cases they were found to be similar [35]–[37], whereas Ntoukakis *et al.* [28] found Pto to be much more active than Fen, with Fen appearing almost kinase inactive. We found that the most likely explanation for these divergent results is the different buffer conditions under which the *in vitro* assays were performed, in particular the pH-dependent availability of Mn.

To assess the activity of both kinases independently of arbitrary buffer conditions, we employed the phospho-specific ProQ stain to determine autophosphorylation levels of both proteins when expressed in *E. coli*. Similar to the results obtained *in vitro* at pH 6.8, Fen appears to be a slightly more active kinase than Pto. At the very least, these results demonstrate that Pto does not have intrinsically higher kinase activity than Fen and that the relative activities are dependent on the buffer conditions used in the assay. We believe that the *in vivo* kinase activities observed in *E. coli* better reflect the kinase activities in plant cells, suggesting that Pto is not a more active kinase than Fen.

To conclusively address the importance of Pto kinase activity in its resistance to AvrPtoB-mediated degradation, we needed a version of Pto that is kinase inactive, but still capable of activating ETI in response to AvrPtoB. We took advantage of Pto(G50S) that confers resistance to AvrPto in tomato. Because G50 is an invariant residue in protein kinases and is involved in ATP binding, we suspected it would have little or no kinase activity. In fact, we were unable to detect any activity either *in vitro* or in *E. coli*. Pto(G50S) was able to activate programmed cell death in *N. benthamiana* in response to AvrPtoB as effectively as wild-type Pto. Together with Xiao *et al.* [40], this result demonstrates that Pto autophosphorylation is not required for activation of ETI in response to either AvrPto or AvrPtoB. The possibility remains that Pto transphosphorylation by another Pto family member as recently proposed [42] is required for the most efficient ETI response.

In retrospect, the model that Pto recalcitrance to AvrPtoB E3 ligase activity is due to phosphorylation of the E3 ligase by Pto is inconsistent with several observations. Specifically, expression of E3 ligase-deficient versions of AvrPtoB in *N. benthamiana* is known to trigger Fen-mediated ETI that can be suppressed by the active E3 ligase domain [22], [24]. In addition, co-expression of Pto with AvrPtoB in *N. benthamiana* does not alone activate ETI, but additionally requires additional expression of tomato Prf [33]. However, if Pto inactivates AvrPtoB E3 ligase activity by phosphorylating the E3 ligase domain, then co-expression of Pto with AvrPtoB in *N. benthamiana* should result in an E3 ligase-inactive AvrPtoB, which in turn should activate Fen-mediated ETI even in the absence of tomato Prf. However, this is not the case [33], which undermines the need to postulate that Pto phosphorylation of AvrPtoB plays a role in its recalcitrance to AvrPtoB E3 ligase activity [28].

In summary, our findings demonstrate that, in contrast to Fen, Pto evolved the capacity to not only bind the E3 ligase-proximal FID of AvrPtoB but additionally to bind the distal N-terminal PID. Interaction with this latter domain is what allows Pto to escape degradation and activate ETI in response to AvrPtoB. Because a kinase-inactive version of Pto is fully capable of activating ETI, phosphorylation of AvrPtoB to inactivate the E3 ligase activity is not a prerequisite for Pto-mediated immunity.

## Materials and Methods

### Plant material

*Solanum lycopersicum* cv. Rio-Grande (RG) and *Nicotiana benthamiana* (accession Nb-1; [43]) plants were grown in a greenhouse with 16 hr light/8 hr dark, 65% humidity and a temperature of 24°C during daylight and 22°C at night. Seeds were germinated on trays and plants were transferred to larger pots 2 weeks post germination.

### Plant inoculations

*Pseudomonas syringae* pathovar *tomato* DC3000 with deletions in the *avrPto* and *avrPtoB* genes (DC3000Δ*avrPto*Δ*avrPtoB*; [44]) was transformed with different variants of AvrPtoB under control of a *Pst hrp*-inducible promoter in pCPP5372 [25]. Bacteria were grown on Kings B solid medium containing 100 µg/mL rifampicin and 10 µg/mL gentamicin at room temperature for 24 hr. The bacteria were scraped from the plates using sterile spatula, resuspended in sterile 10 mM MgCl_2_ and the OD_600_ was adjusted to 0.2 (corresponding to ∼5×10^8^ CFU/mL). Serial dilutions in 10 mM MgCl_2_ were made to generate bacterial suspensions of 5×10^4^ CFU/mL. Tomato plants were infiltrated by submersion of the aerial parts of the plant in the bacterial suspensions containing 0.002% Silwet L-77 and applying a vacuum of −80 kPa for 2 min. Plants were allowed to dry lying on their side and transferred into a growth chamber. Disease development was monitored for up to one week after infiltration.

### Cloning

All enzymes and reagents used for cloning were purchased from New England Biolabs (Ipswich, MA, USA) unless otherwise noted. For Gateway entry vectors, the open-reading frames (ORFs) were amplified with or without a stop codon using Phusion DNA polymerase and blunt-end ligated into the SmaI site of pJM51 or pJLSmart. Following sequence confirmation, recombination reactions were performed using Gateway LR Clonase II (Life Technologies, Carlsbad, CA, USA) following the manufacturer's instructions to transfer the ORFs into Gateway compatible destination vectors. Recombination sites were confirmed by sequencing.

The yeast two-hybrid vectors used in this study (pEG202 and pJG4-5) are not Gateway compatible. Consequently, ORFs for expression in yeast were amplified using gene-specific primers adding EcoRI sites at the 5′ and 3′ ends and the PCR products were digested with EcoRI and ligated into pEG202 or pJG4-5. Orientation of the insert was confirmed by colony PCR, and the complete ORF of the inserted gene was confirmed by sequencing.

*In vitro* mutagenesis was performed following standard protocols (Stratagene QuikChange site directed mutagenesis kit). In brief, complementary oligos were designed for both strands containing the desired nucleotide changes flanked by at least 15 nucleotides perfectly matching the template DNA. These oligos were used in a 15- to 18-cycle PCR with Phusion DNA polymerase and 50 ng of a vector containing the original (unchanged) DNA sequence. The PCR product was subjected to a DpnI digest to remove template plasmid and 2–5 µl of the resultant solution were used to transform *E. coli* DH5α.

Fusion protein ORFs were generated by fusion PCRs. First, PCR reactions were performed using oligos that added 15–25 nt of additional sequence homologous to the ‘other’ side of the desired fusion border to the 3′ (for the N-terminal fusion part) or 5′ (for the C-terminal fusion part) end of the PCR product. These PCR products were purified and used as templates in a PCR using only the oligos corresponding to the 5′ (forward oligo) and 3′ (reverse oligo) of the desired final fusion ORF, generating the full length fusion. Full-length PCR products were ligated into the different plasmids as described above and confirmed by sequencing.

Details of oligonucleotides (**Table S1**), vectors (**Table S2**), and constructs (**Table S3**) used in this work are provided in the supplemental information. Complete sequences as well as Gene Construction Kit (GCK) format vector maps of all constructs generated in this work are available upon request.

### *In vitro* ubiquitin ligase assay

Ubiquitin ligase assays were performed as described previously [26]. In brief, 1 µg of purified GST:AvrPtoB was added to a solution containing 50 nM E1 (UBE1), 100 nM E2 (UbcH5a), 10 µg Ubiquitin, 2 mM DTT, 5 mM ATP, 5 mM MgCl_2_ and 50 mM Tris at pH 7.5 in a total volume of 30 µl for 2 hrs at 30°C. Reactions were stopped by the addition of 30 µl 2× reducing Laemmli buffer and boiling for 5 min. E1 (#E-304), E2 (#E2-616) and ubiquitin (#U-100) were purchased from Boston Biochemical (Cambridge, MA, USA). Proteins were resolved by SDS-PAGE, transferred to PVDF membranes by Western blotting and detected using monoclonal mouse anti-ubiquitin antibodies (clone P4D1, SC-8017, Santa Cruz Biotechnology, Santa Cruz, CA, USA) at a dilution of 1/5000.

### *In vitro* kinase assays

Kinase assays were performed as described previously [35] and as elaborated upon in the text. Tris-HCl (50 mM) at either pH 6.8 or 7.5 and 10 mM of either MnCl_2_, MgCl_2_ or both were used in the buffers to test for the impact of buffer conditions on kinase activities.

### Pro-Q phospho-protein staining

Proteins were purified from *E. coli* and resolved by SDS-PAGE. Pro-Q staining and Coomassie Silver counter-staining were performed exactly as described by Taylor et al. 2013 [39]. Pro-Q was obtained from Invitrogen Co. (Eugene, OR)

### Yeast two-hybrid assays

The LexA yeast two-hybrid system was used to investigate the interactions of different forms of AvrPtoB with Pto and Fen [22], [24]. ORFs were inserted into the EcoRI sites of pEG202 (bait) or pJG4-5 (prey) vectors and introduced into *Saccharomyces cerevisiae* strain EGY48 containing the reporter plasmid pSH18-34. Transformations were plated onto CM (minus uracil, histidine, tryptophan, UHW) dropout medium containing glucose and single colonies of primary transformants were replica-plated onto fresh CM –UHW/glucose master plates and grown overnight. Subsequently, these colonies were replica-plated onto inductive CM –UHW plates containing raffinose/galactose and 5-bromo-4-chloro-3-indolyl-β-D-galactopyranoside (X-Gal) and staining was monitored for 24 to 48 hrs.

### *Agrobacterium*-mediated transient protein expression

*Agrobacterium tumefaciens* strain GV3101 with helper plasmid pMP90 was transformed with constructs containing the genes to be expressed under control of a CaMV 35S promoter using electroporation. Single colonies were selected and the presence of the correct transgene confirmed by colony PCR. Agrobacteria were grown as a lawn on Luria Bertani medium plates containing appropriate antibiotics for 24 hr at 30°C. Bacteria were scraped from the plates using a sterile spatula and resuspended in sterile infiltration medium (10 mM MgCl_2_, 50 mM MES pH 5.6, 500 µM aceto-syringone) and the OD_600_ determined. Based on the OD, aliquots of the solutions corresponding to a desired density in a desired volume were transferred to fresh tubes, pelleted by centrifugation (2000 rcf, 10 min, RT), decanted and the pellets resuspended in the correct volume of fresh buffer.

Leaves of 4 to 6-week old *N. benthamiana* plants were infiltrated with bacterial suspensions containing an OD_600_ of 0.3 for each construct using a needle-less syringe. Plants were transferred to a growth chamber shelf with no lights and surrounded by shade cloth after infiltration. For analysis of protein expression by Western blotting, samples were collected 48 hr after infiltration. Cell death in the infiltrated areas was monitored for up to one week after infiltration; cell death typically became macroscopically visible 48 hr after infiltration and was completely developed 5 d after infiltration.

## Supporting Information

Figure S1 ***N. benthamiana*** **‘Rsb’ type resistance is triggered by AvrPtoB_1–307_** (A) Different variants of AvrPtoB were expressed in a leaf of *N. benthamiana* by *Agrobacterium*-mediated transient transformation. AvrPtoB_1–307_ was sufficient to elicit ETI-associated cell death. YFP was included as a negative control. (B) Western blot to determine expression levels for the different variants of AvrPtoB. Fusion proteins were detected using anti-c-Myc-HRP rabbit polyclonal antibody (SC-789, Santa Cruz Biotech., Santa Cruz, CA, USA). (C) A mutation deactivating the PID in AvrPtoB is sufficient to suppress endogenous *N. benthamiana* ‘Rsb’ type resistance. AvrPtoB variants were expressed transiently as in (A).
(PDF)

Figure S2 **Comparison of Fen and Pto kinase activities using protocol of Ntoukakis et al.,** [28], **and discoloration of Mn^2+^-containing Tris kinase buffer is pH dependent**. (A) *In vitro* kinase assay for tomato Pto and Fen at pH 7.5. Maltose-binding protein (MBP) fusions, MBP:Pto, MBP:Fen, or MBP alone were purified from *E. coli* and subjected to an *in vitro* kinase assay. At this high pH, Pto was a more active kinase than Fen. However, presence of 10 mM MnCl_2_ in the kinase buffer caused a brown discoloration under these conditions. CBB, Coomassie Brilliant Blue. (B) Kinase buffers containing 50 mM Tris-HCl and 1 mM DTT at the indicated pH were prepared and pH was confirmed by both pH meter and pH test strips. Upon addition of 10 mM MnCl_2_, a brown discoloration was observed in all buffers above pH 7.0 that increased in intensity with increasing pH.
(PDF)

Figure S3 **A summary of the proximity model.** The proximity to the E3 ligase of Pto binding and not the ability of Pto to phosphorylate AvrPtoB determines whether or not it escapes E3 ligase-mediated ubiquitination/degradation. Pto bound at the PID escapes ubiquitination (shown as an X) whereas Fen and Pto bound to the FID are ubiquitinated/degraded (shown as the red arrow and poly-ubiquitination (Ub) of the kinases). Pto bound at the PID activates effector-triggered immunity (ETI).
(PDF)

Table S1 **Oligonucleotides used in this work.**
(PDF)

Table S2 **Vectors used for plasmid generation.**
(PDF)

Table S3 **Constructs generated for this work.**
(PDF)
