# Supplementary figures and images for: Pto Kinase Binds Two Domains of AvrPtoB and Its Proximity to the Effector E3 Ligase Determines if It Evades Degradation and Activates Plant Immunity
Source: PLoS Pathog. 2014 Jul 24;10(7):e1004227. doi: 10.1371/journal.ppat.1004227 (PMC4110037; doi:10.1371/journal.ppat.1004227)

# Figure S1

A

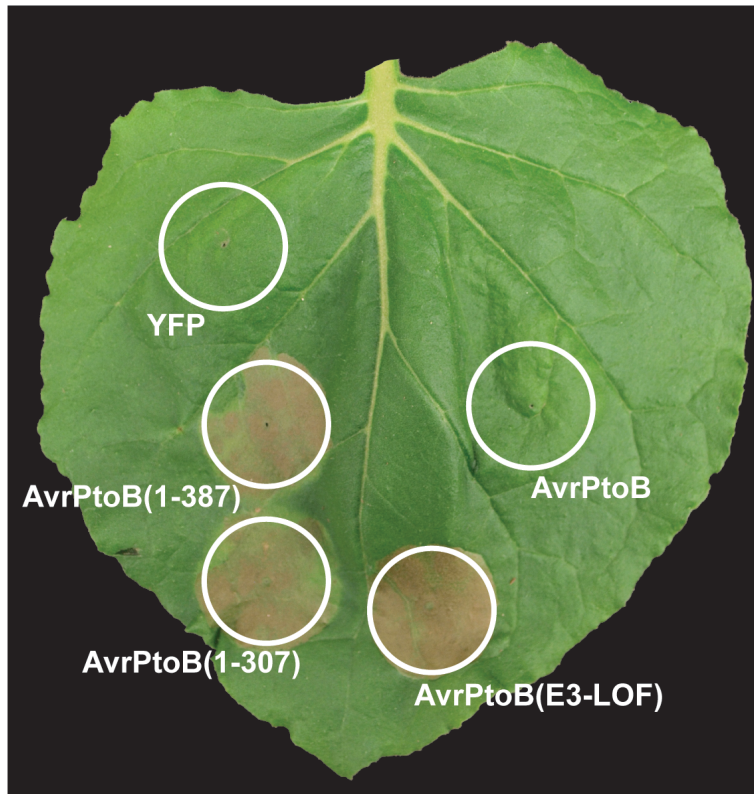

B

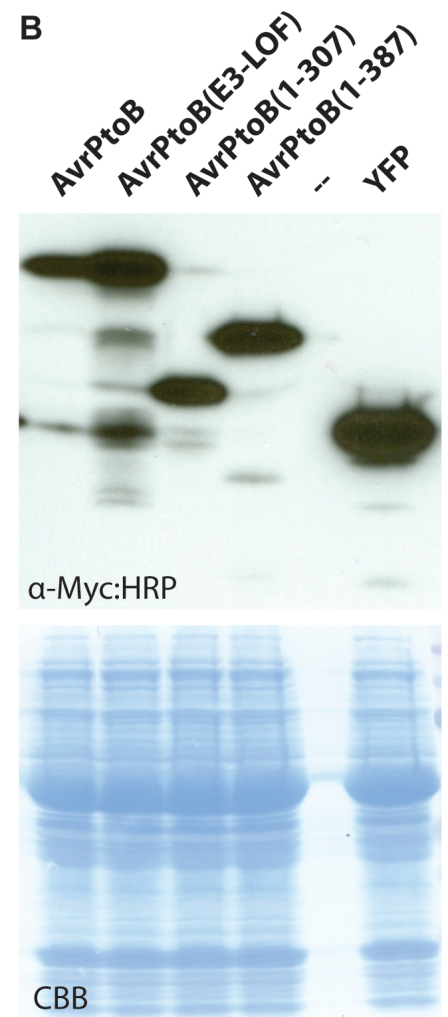

C

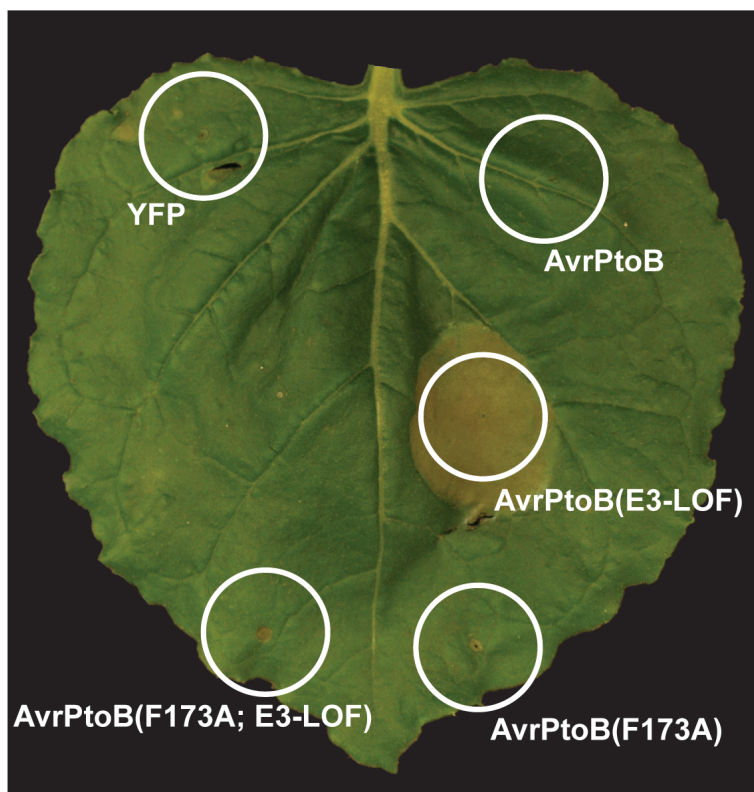

Supplement: Figure S1 — N. benthamiana ‘Rsb’ type resistance is triggered by AvrPtoB1–307 (A) Different variants of AvrPtoB were expressed in a leaf of N. benthamiana by Agrobacterium-mediated transient transformation. AvrPtoB1–307 was sufficient to elicit ETI-associated cell death. YFP was included as a negative control. (B) Western blot to determine expression levels for the different variants of AvrPtoB. Fusion proteins were detected using anti-c-Myc-HRP rabbit polyclonal antibody (SC-789, Santa Cruz Biotech., Santa Cruz, CA, USA). (C) A mutation deactivating the PID in AvrPtoB is sufficient to suppress endogenous N. benthamiana ‘Rsb’ type resistance. AvrPtoB variants were expressed transiently as in (A). (PDF) [file ppat.1004227.s001.pdf]

**A**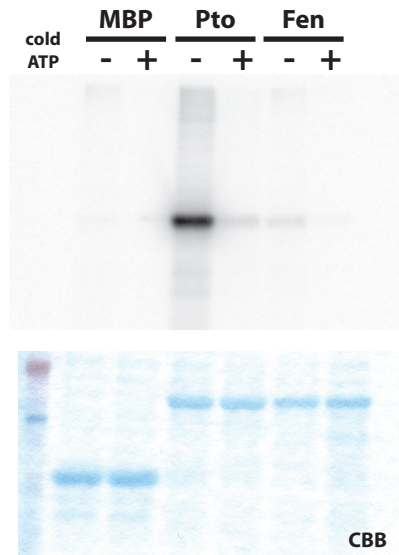**B**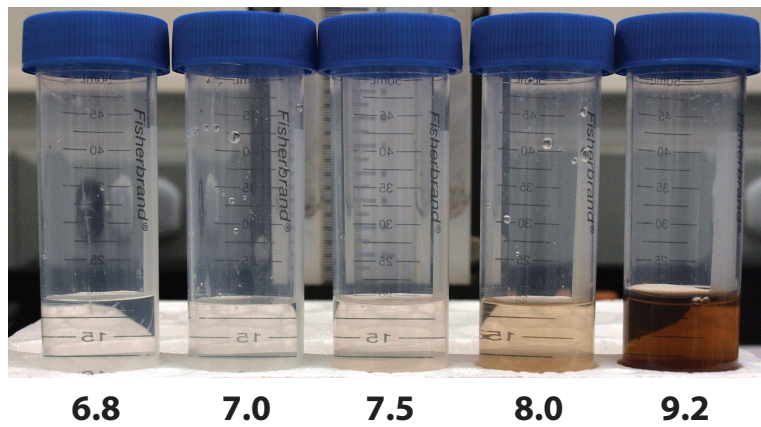

Supplement: Figure S2 — Comparison of Fen and Pto kinase activities using protocol of Ntoukakis et al., [28], and discoloration of Mn2+-containing Tris kinase buffer is pH dependent. (A) In vitro kinase assay for tomato Pto and Fen at pH 7.5. Maltose-binding protein (MBP) fusions, MBP:Pto, MBP:Fen, or MBP alone were purified from E. coli and subjected to an in vitro kinase assay. At this high pH, Pto was a more active kinase than Fen. However, presence of 10 mM MnCl2 in the kinase buffer caused a brown discoloration under these conditions. CBB, Coomassie Brilliant Blue. (B) Kinase buffers containing 50 mM Tris-HCl and 1 mM DTT at the indicated pH were prepared and pH was confirmed by both pH meter and pH test strips. Upon addition of 10 mM MnCl2, a brown discoloration was observed in all buffers above pH 7.0 that increased in intensity with increasing pH. (PDF) [file ppat.1004227.s002.pdf]

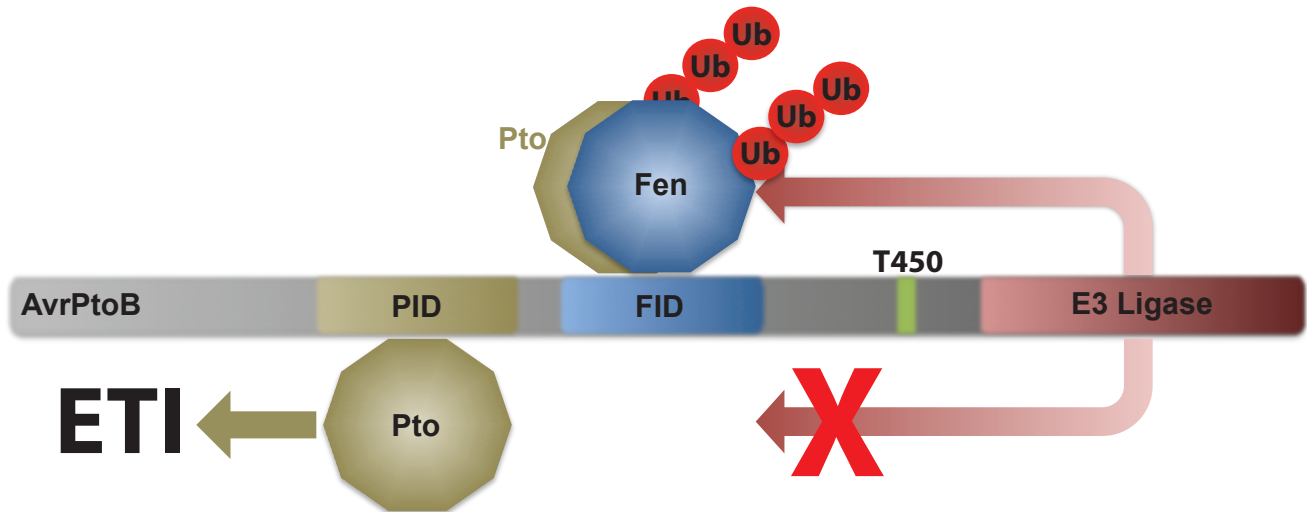

Supplement: Figure S3 — A summary of the proximity model. The proximity to the E3 ligase of Pto binding and not the ability of Pto to phosphorylate AvrPtoB determines whether or not it escapes E3 ligase-mediated ubiquitination/degradation. Pto bound at the PID escapes ubiquitination (shown as an X) whereas Fen and Pto bound to the FID are ubiquitinated/degraded (shown as the red arrow and poly-ubiquitination (Ub) of the kinases). Pto bound at the PID activates effector-triggered immunity (ETI). (PDF) [file ppat.1004227.s003.pdf]
