# Supplementary material for: Pto Kinase Binds Two Domains of AvrPtoB and Its Proximity to the Effector E3 Ligase Determines if It Evades Degradation and Activates Plant Immunity
Source: PLoS Pathog. 2014 Jul 24;10(7):e1004227. doi: 10.1371/journal.ppat.1004227 (PMC4110037; doi:10.1371/journal.ppat.1004227)
Supplement: Table S1 — Oligonucleotides used in this work. (PDF) [file ppat.1004227.s004.pdf]

Table S1
Oligonucleotides used in this work

Design.	sequence	use	features
oJM08	atggcgggtatcaatagagcg	fwd oligo for all AvrPtoB blunt end clonings	Contains Start codon
oJM09	tcaggggactattctaaaagc	rev oligo to clone FL AvrPtoB blunt end	contains STOP codon
oJM154	tggggactattctaaaagc	rev oligo to clone AvrPtoB entry vectors without STOP codon	removes STOP codon and corrects frame
oJM83	gggaattcgcgggtatcaatagagcg	fwd oligo for all Y2H AvrPtoB clonings	Adds 5' EcoR I for in frame clonings into pEG202, pJG4-5
oJM130	gcgaattctcaggggactattctaaaagc	rec oligo for all Y2H AvrPtoB clonings	Adds 3' EcoR I for in frame clonings into pEG202, pJG4-5
oJM53	ctacatgtctttcaagggccgt	rev oligo to clone AvrPtoB(1-307) no STOP entry vectors	removes STOP codon and corrects frame
oJM54	ccacccgcaatcgtgttgc	rev oligo to clone AvrPtoB(1-387) no STOP entry vectors	removes STOP codon and corrects frame
oJM14	ttcagtcagctgcgcgcaatttcgaaggccg	fwd oligo in vitro mutagenesis to generate AvrPtoB(T450A)	
oJM15	cggccttcgaaattgcgcgcagctgactgaa	rev oligo in vitro mutagenesis to generate AvrPtoB(T450A)	
oJM134	caattcagtcagctgcgcgatatctcgaaggccgatgctgaatctg	fwd oligo in vitro mutagenesis to generate AvrPtoB(T450D)	
oJM135	cagattcagcatcggccttcgagatatcgcgcagctgactgaattg	rev oligo in vitro mutagenesis to generate AvrPtoB(T450D)	
oSS77	gcagaatgtggcaattaacccaagtatcg	fwd oligo in vitro mutagenesis to generate AvrPtoB(G325A)	
oSS78	cgatacttgggttaattgccacattctgc	rev oligo in vitro mutagenesis to generate AvrPtoB(G325A)	
oJM433	aattggacatagtgtctttggga	fwd oligo in vitro mutagenesis to generate Pto(G50S)	
oJM434	tcccaaagacactatgtccaatt	rev oligo in vitro mutagenesis to generate Pto(G50S)	
oJM189	atgggaagcaagtattccaa	fwd oligo for Fen entry vector clonings	Contains Start codon
oJM190	attcaggatcatcttgaat	rev oligo to clone Fen no STOP entry vectors	removes STOP codon and corrects frame
oJM199	tcattcaggatcatcttgaat	rev oligo to clone Fen entry vectors with STOP	contains STOP codon
oJM163	atgggaagcaagtattctaa	fwd oligo for Pto entry vector clonings	Contains Start codon
oJM165	aaataacagactcttggaga	rev oligo to clone Pto no STOP entry vectors	removes STOP codon and corrects frame
oJM164	ttaaataacagactcttgga	rev oligo to clone Pto entry vectors with STOP	contains STOP codon
oJM109	cttcgtcgagcggttcacatgccggagcgggaggattac	fwd oligo to generate AvrPtoB PID-E3 ligase fusions	creates AvrPtoB fusion ORF fusing aa 200 to aa 388
oJM108	gtaatcctcccgctccggcatgtgaaccgctcgacgaag	rev oligo to generate AvrPtoB PID-E3 ligase fusions	creates AvrPtoB fusion ORF fusing aa 200 to aa 388

Table S1
All oligonucleotides were ordered from Integrated DNA Technologies (Coralville, IA , USA)
