# Supplementary material for: Pto Kinase Binds Two Domains of AvrPtoB and Its Proximity to the Effector E3 Ligase Determines if It Evades Degradation and Activates Plant Immunity
Source: PLoS Pathog. 2014 Jul 24;10(7):e1004227. doi: 10.1371/journal.ppat.1004227 (PMC4110037; doi:10.1371/journal.ppat.1004227)
Supplement: Table S2 — Vectors used for plasmid generation. (PDF) [file ppat.1004227.s005.pdf]

Table S2
Vectors used for plasmid generation

Vector	purpose	Source
pGEX-4T	N-terminal GST fusions for expression in / purification from *E. coli*	GE Healthcare Life Sciences, Pittsburg, PA, USA
pMAL-c2	N-terminal MBP fusions for expression in / purification from *E. coli*	New England Biolabs, Ispwich, MA, USA
pEG202	Yeast-two hybrid bait vector	Golemis et al. 2008
pJG4-5	Yeast-two hybrid prey vector	Golemis et al. 2008
pJLSmart	GW entry vector for expression in eukaryotes; N- and C-terminal fusions	Mathieu et al. 2007
pJM51	modified pJLSmart GW entry vector: contains Shine Dalgarno sequence for expression in prokaryotes	Cheng et al. 2011
pGWB417	GW destination vector for transient protein expression in N benth. Adds C-terminal 4x Myc tag	Nakagawa et al. 2007
pCPP5372	GW destination vector for effector expression in *Pst*. Contains hrp promoter and C-terminal HA tag.	Oh et al. 2007
pBTEX:PRF	Co-expression of tomato PRF with Pto in N benth.	Du et al. 2012
pMAL-Pto	Expression of Pto in E. coli	Loh et al. 1995
pMAL-Fen	Expression of Fen in E. coli	Loh et al. 1995

Table S2
