# Supplementary material for: Pto Kinase Binds Two Domains of AvrPtoB and Its Proximity to the Effector E3 Ligase Determines if It Evades Degradation and Activates Plant Immunity
Source: PLoS Pathog. 2014 Jul 24;10(7):e1004227. doi: 10.1371/journal.ppat.1004227 (PMC4110037; doi:10.1371/journal.ppat.1004227)
Supplement: Table S3 — Constructs generated for this work. (PDF) [file ppat.1004227.s006.pdf]

Table S3
Constructs generated in the course of this work

Designation	Vector	Insert	comments
pJM99	pJG4-5	PID:E3 fusion	genrated by fusion PCR
pJM107	pJG4-5	PID:E3-LOF fusion	genrated by fusion PCR
pJM128	pJG4-5	AvrPtoB[(T450A)]	in vitro mutagenesis
pJM64	pJM51	AvrPtoB	PCR cloning into Sma I
pJM70	pJM51	AvrPtoB[E3-LOF]	in vitro mutagenesis
pSS66	pJM51	AvrPtoB[(F173A)	in vitro mutagenesis
pSS67	pJM51	AvrPtoB[(F173A) E3-LOF]	in vitro mutagenesis
pJM122	pJM51	AvrPtoB[(T450A)]	in vitro mutagenesis
pJM123	pJM51	AvrPtoB[(T450D)]	in vitro mutagenesis
pJM65	pJM51	AvrPtoB[1-307]	PCR cloning into Sma I
pJM66	pJM51	AvrPtoB[1-387]	PCR cloning into Sma I
pPB04	pJLSmart	Pto	PCR cloning into Sma I
pJM212	pJLSmart	Fen	PCR cloning into Sma I
pJM482	pJLSmart	Pto(G50S)	in vitro mutagenesis
pJM371	pJLSmart	YFP	PCR cloning into Sma I
pJM69	pCPP5372	AvrPtoB	LR recombination
pJM155	pCPP5372	AvrPtoB[E3-LOF]	LR recombination
pJM292	pCPP5372	AvrPtoB[(F173A)	LR recombination
pJM293	pCPP5372	AvrPtoB[(F173A) E3-LOF]	LR recombination
pJM145	pCPP5372	AvrPtoB[(T450A)]	LR recombination
pJM146	pCPP5372	AvrPtoB[(T450D)]	LR recombination
pJM306	pGWB417	AvrPtoB	LR recombination
pJM336	pGWB417	AvrPtoB[E3-LOF]	LR recombination
pJM349	pGWB417	AvrPtoB[(F173A)	LR recombination
pJM414	pGWB417	AvrPtoB[(F173A) E3-LOF]	LR recombination
pJM352	pGWB417	AvrPtoB[1-307]	LR recombination
pJM353	pGWB417	AvrPtoB[1-387]	LR recombination
pJM307	pGWB417	Pto	LR recombination
pJM311	pGWB417	Fen	LR recombination
pJM493	pGWB417	Pto(G50S)	LR recombination
pJM374	pGWB417	YFP	LR recombination
pJM68	pGEX-4T	AvrPtoB	PCR cloning into EcoR I / Not I
pJM392	pGEX-4T	AvrPtoB[E3-LOF]	PCR cloning into EcoR I
pJM393	pGEX-4T	AvrPtoB[(T450A)]	PCR cloning into EcoR I
pJM394	pGEX-4T	AvrPtoB[(T450D)]	PCR cloning into EcoR I
pJM483	pMAL-c2	Pto(G50S)	PCR cloning into EcoR I

Table S3
Complete sequences and vector maps in Gene Construction Kit (.GCK) format for all constructs are available upon request.
